# Conserved domains can be found across distinct phage defence systems

**DOI:** 10.1111/mmi.15047

**Published:** 2023-03-09

**Authors:** Giuseppina Mariano, Tim R. Blower

**Affiliations:** ^1^ Microbes in Health and Disease Theme, Newcastle University Biosciences Institute Newcastle University Newcastle upon Tyne UK; ^2^ Department of Biosciences Durham University Durham UK

**Keywords:** antiphage proteins, bacterial defence, conserved protein domains, phage resistance

## Abstract

Bacteria are continuously exposed to predation from bacteriophages (phages) and, in response, have evolved a broad range of defence systems. These systems can prevent the replication of phages and other mobile genetic elements (MGE). Defence systems are often encoded together in genomic loci defined as “defence islands”, a tendency that has been extensively exploited to identify novel antiphage systems. In the last few years, >100 new antiphage systems have been discovered, and some display homology to components of the immune systems of plants and animals. In many instances, prediction tools have found domains with similar predicted functions present as different combinations within distinct antiphage systems. In this Perspective Article, we review recent reports describing the discovery and the predicted domain composition of several novel antiphage systems. We discuss several examples of similar protein domains adopted by different antiphage systems, including domains of unknown function (DUFs), domains involved in nucleic acid recognition and degradation, and domains involved in NAD+ depletion. We further discuss the potential evolutionary advantages that could have driven the independent acquisition of these domains by different antiphage systems.

## Graphical abstract

Bacteria have evolved diverse defence systems to prevent predation from bacteriophages. Recent advances in the investigation of the bacteria‐bacteriophage arms race have highlighted how defence systems often co‐opt the same protein domains in a variety of combinations. This mix‐and‐match of similar defence domains supports the rapid generation of a diverse arsenal of defence systems. These can then be swiftly deployed against a broad spectrum of phages, potentially minimising the risk of phages developing counter‐resistance.

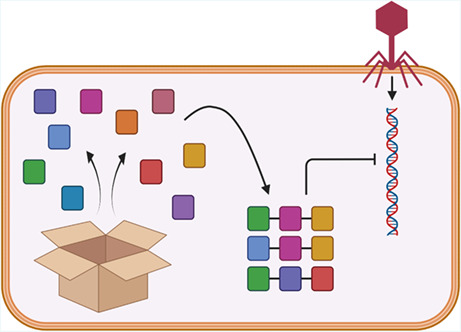

## 1 INTRODUCTION

Bacteriophages (phages) and bacteria coexist in every niche, where phages are estimated to outnumber bacteria by 10‐fold. This strong selective pressure has led bacteria to evolve defence strategies that prevent phage replication and spread. In response, phages have adapted to these defence pathways, evolving counter‐strategies and generating a continuous arms race that, over time, has shaped both bacterial and viral populations. Earlier research efforts on phage defence have focused on the investigation of restriction‐modification (R‐M) systems, abortive infection systems and CRISPR‐Cas and, later, on the Bacteriophage Exclusion (BREX) system and defence island system associated with restriction‐modification (DISARM) (Barrangou et al., 2007; Goldfarb et al., 2015; Kinch et al., 2005; Ofir et al., 2018). However, in recent years it has become increasingly clear that the diversity of phage systems is much higher than expected.

Antiphage systems are generally clustered in genomic loci, defined as “defence islands” (Makarova et al., 2011; Picton et al., 2021). Defence islands are often encoded within various MGEs such as prophages, phage‐inducible chromosomal islands (PICIs) and plasmids (Fillol‐Salom et al., 2022; Hochhauser et al., 2022; Ibarra‐Chávez et al., 2022; Vassallo et al., 2022). MGEs often drive the mobilisation and the resulting distribution of antiphage systems across bacterial species. Recent bioinformatic studies have successfully employed a “guilt by association” approach to identify novel operons involved in defence against phages based on their frequent co‐localisation with known antiphage systems. Greater than 100 new antiphage systems have been discovered in recent years, though their association with defence islands and abundance in bacterial genomes can be variable (Doron et al., 2018; Gao et al., 2020; Millman et al., 2020, 2022; Rousset et al., 2022; Vassallo et al., 2022). The discovery of such an abundance of novel defence systems has been followed by experimental verification of their involvement in bacterial immunity, prediction of protein domains and 3D folds for each component of the novel systems and, in some cases, determination of their mechanism of action.

These studies provided the unprecedented revelation that several antiphage systems exhibit homology to components of the known innate immune system of plants and animals, and this has now been reversed, allowing the prediction of immunity genes within eukaryotes (Bernheim et al., 2021; Cury et al., 2022; Govande et al., 2021; Johnson et al., 2022; Millman et al., 2022). Furthermore, protein domain prediction has highlighted that components of various antiphage systems have acquired domains of similar function, and these domains appear in various combinations (Doron et al., 2018; Gao et al., 2020; Millman et al., 2022; Rousset et al., 2022; Vassallo et al., 2022).

This Perspective Article reviews the range of protein domains that distinct antiphage systems have acquired. We begin by reviewing the antiphage systems that share a common domain. We then discuss the advances in determining the details of their mode of action and the role of the shared domains in the defence process where established. Finally, we discuss the evolutionary implication of acquiring common domains by different antiphage systems and the potential advantages that could have driven their independent acquisition.

### 1.1 CONSERVED DOMAINS IN ANTIPHAGE PROTEINS

#### 1.1.1 DUF262 and DUF1524

DUF262 and DUF1524 were first found in type IV restriction‐modification (R‐M) system GmrSD. GmrSD enzymes exhibit specificity for glucosyl‐5‐hydroxymethyl cytosine (glc‐5hmC) modifications and are active against T‐even phages. Whilst the first GmrSD example discovered was constituted by two distinct proteins, in most cases, GmrSD homologues are found as a single polypeptide (Machnicka et al., 2015).

DUF262 is found to be associated with the GmrS component and is typically related to the ParB/Srx‐like fold. Bioinformatic analysis and modelling showed that the GmrS DUF262 contains a conserved (I/V)‐D‐G‐Q‐Q‐R domain that forms an NTP binding pocket and is likely responsible for NTPase activity. Conversely, GmrD contains the DUF1524 domain, part of the His‐Mer finger endonucleases superfamily. Accordingly, GmrD modelling showed a fold similar to HNH nucleases and the presence of a conserved DHIYP domain (Machnicka et al., 2015). In vitro testing of the Eco94GmrSD homologue showed that it digests T4 DNA in the presence of Mg^2+^ or Mn^2+^ but not with other divalent cations. Additionally, ATP and TTP promote Eco94GmrSD activity, whilst no changes were detected in the presence of CTP and GTP (He et al., 2015).

Recently, a GmrSD homologue with low sequence similarity, BrxU, was found to be associated with a BREX system within a defence island (Picton et al., 2021). Despite the low sequence similarity BrxU possesses both the DUF262 and DUF1524 domains. Picton et al. showed that, unlike GmrSD, BrxU activity is indiscriminately promoted by all NTPs, dNTPs and a wide set of divalent metal cations. BrxU was also shown to have a more relaxed specificity for various types of cytosine modifications (5mC, 5hmC or glc‐5hmC) compared to GmrSD (Picton et al., 2021). Interestingly, Picton et al., demonstrated that BrxU and the associated BREX system concerted action provides complementary resistance to modified and non‐modified phages (Picton et al., 2021). The BrxU structure represents the first solved structure of the GmrSD family and intriguingly showed that BrxU proteins normally exist as dimers. Upon NTP binding, BrxU transitions to a monomeric form and it is hypothesised that, following NTP hydrolysis, BrxU protomers re‐associate to form a dimer before recognising modified DNA and completing its cleavage (Picton et al., 2021).

SspE proteins, part of SspABCD–SspE phosphorothioation‐sensing bacterial defence systems, also contain predicted DUF262 and DUF1524 domains. Nevertheless, the reported role of SspE appears distinct from that observed for GrmSD and BrxU (Gao et al., 2022; Xiong et al., 2020). The SspE structure highlighted the DUF262 retains the conserved DGQQR motif in the nucleotide‐binding pocket (Gao et al., 2022; Xiong et al., 2020). As per BrxU, SspE DUF262 can hydrolyse GTP, CTP, UTP and ATP indiscriminately. Gao et al., further demonstrated that an N‐terminal hydrophobic patch on SspE surface recognises the 5′‐C_PS_CA‐3′ PT modification and triggers NTPase activity. Fluorescence resonance energy transfer (FRET) measurements demonstrated that DUF262 NTPase activity, upon PT recognition, triggers a conformational change that activates the DUF1524 domain (Gao et al., 2022). Whereas DUF1524 of GmrSD and BrxU causes cleavage of modified DNA, within SspE the DUF1524 promotes DNA nicking (Gao et al., 2022; Xiong et al., 2020).

Finally, DUF262 domains were also found in recently‐identified systems PD‐T4‐2, Menshen and Dazbog (Millman et al., 2022; Vassallo et al., 2022). None of these systems, however, contains a DUF1524 domain. Mutation or deletion of genes harbouring DUF262 abolished phage resistance for both Menshen and Dazbog. In Menshen, the DUF262 gene is associated with a predicted OLD nuclease, and thus, its role could include NTPase activity to regulate its associated nuclease, similarly to other DUF262 harbouring proteins. Furthermore, a high‐throughput analysis of phage factors that drive sensitivity to antiphage systems highlighted that the DUF262 in Dazbog likely allows recognition of methylated DNA (Millman et al., 2022; Stokar‐Avihail et al., 2022). Future work will confirm whether, in these systems, the role of the DUF262 component is similar to that observed for GmrSD and BrxU, and establish their nucleotide selectivity.

#### 1.1.2 Domains involved in DNA‐binding and DNA‐degradation

The association of DUF262 and DUF1524, a trait shared by GmrSD, BrxU and SspE, represents a well‐characterised example wherein one or more proteins that form either a whole or component part of an antiphage system, harbour a domain that is involved in binding, modification or degradation of nucleotides and nucleic acids. Indeed, domain prediction approaches employed in recent studies that reported the discovery of new antiphage systems have highlighted the high frequency of occurrence of such domains (Doron et al., 2018; Gao et al., 2020; Millman et al., 2022; Rousset et al., 2022; Vassallo et al., 2022).

In some cases, one or more components of different antiphage systems possess predicted domains that share the same PFAM identifier. Therefore, a clear link between their independent acquisition by distinct antiphage systems is readily detectable (Table 1). For these domains, it is easy to speculate that they may provide an efficient strategy to arrest phage infection, leading to their independent acquisition by different antiphage systems. For example, the GajA component of the Gabija system is a DNA‐nicking endonuclease, and its ATPase domain regulates GajA activity through ATP (and GTP)‐mediated inhibition (Cheng et al., 2021, 2022). PFAM domains PF13175 and PF3245, both with predicted AAA+ ATPase activity, were associated with GajA but are also found in several other antiphage systems, such as PARIS subtypes, PD‐T4 and Old‐tin (Doron et al., 2018; Rousset et al., 2022; Vassallo et al., 2022) (Table 1). The role and regulation of PF13175 and PF3245 in other antiphage systems remain to be established. GajA also presents a topoisomerase‐primase (TOPRIM) domain of OLD family nucleases (PF20469) at its C‐terminus, which is responsible for its nicking activity acting on both phage and chromosomal DNA (Cheng et al., 2021). Domains of the same family are also associated with one of the components of the Menshen system and in the Retron+TOPRIM system, but their roles remain unknown to date (Gao et al., 2020; Millman et al., 2022). An OLD nuclease family TOPRIM domain was also predicted with a weaker score in the AriB member of PARIS systems (Rousset et al., 2022). Furthermore, GajB exhibits a UvrD‐like helicase (PF00580 and PF13361) and was recently shown to bind to DNA termini produced during replication and recombination events and hydrolyses (d)ATP or (d)GTP to promote GajA activity (Cheng et al., 2022; Doron et al., 2018). PF00580 and PF13361 were also detected in PARIS1 and the Helicase+ DUF2290 antiphage systems (Doron et al., 2018; Rousset et al., 2022) but in this cases, their role was not explored.

**TABLE 1:** List of predicted PFAM domains shared by distinct antiphage systems.

PFAM domain identifier	Predicted function	Antiphage systems with PFAM domain	Citation	Experimentally tested function
PF00270	DEAD and DEAH box helicases	Hachiman Druantia Helicase+DUF2290	Doron et al. (2018), Rousset et al. (2022)	N/A
PF00271	DEAD/H helicases	Druantia Zorya I Hachiman DISARM AbpA/AbpB system	Yasui et al. (2014), Doron et al. (2018)	N/A
PF01844	HNH‐endonuclease	Septu Zorya II	Doron et al. (2018)	N/A
PF08878	CAP4‐SAVED	Hachiman HP + HP + HP AbpA/AbpB system	Yasui et al. (2014), Doron et al. (2018), Rousset et al. (2022)	
PF13175	AAA+ ATPase domain	PARIS 1 PARIS 2 Old‐tin Gabija PD‐T4‐4	Doron et al. (2018), Rousset et al. (2022), Vassallo et al. (2022)	N/A
PF13245	AAA+ ATPase domain	PARIS 1 PARIS 2 Helicase+DUF2290 Old‐tin Gabija TIR‐NLR	Doron et al. (2018), Rousset et al. (2022)	N/A
PF13304	AAA+ ATPase domain	PARIS1 PARIS 2 Septu Old‐tin	Doron et al. (2018), Rousset et al. (2022)	N/A
PF13555	AAA+ ATPase domain	Wadjet PARIS1 PARIS 2 Old‐tin TIR‐NLR	Doron et al. (2018), Rousset et al. (2022)	N/A
PF09820	AAA+ ATPase domain	Radar (RdrA) PARIS1 Old‐tin	Doron et al. (2018), Rousset et al. (2022), Duncan‐Lowey et al. (2023)	In RADAR, RdrA hydrolyses ATP to activate RdrB
PF00580	UvrD‐Helicase	PARIS 1 Helicase+DUF2290 Gabija	Doron et al. (2018), Rousset et al. (2022)	In GajB: sensing of DNA termini and hydrolysis of (d)ATP or (d)GTP
PF13361	UvrD‐Helicase	Helicase+DUF2290 Gabija	Doron et al. (2018), Rousset et al. (2022)	In GajB: sensing of DNA termini and hydrolysis of (d)ATP or (d)GTP
PF02463	SMC ATPase	Lamassu PARIS1 PARIS2 Old‐tin Wadjet	Doron et al. (2018), Rousset et al. (2022), Millman et al. (2022), Deep et al. (2022)	Hypothetical DNA binding for recognition of phage invasion
PF00176	Snf2‐Helicase	Zorya I Helicase+DUF2290 DISARM	Ofir et al. (2018), Doron et al. (2018), Rousset et al. (2022)	N/A
DUF262	NTPase	GmrSD BrxU SspABCD‐SspE Menshen Dazbog PD‐T4‐2	Machnicka et al. (2015), Picton et al. (2021), Millman et al. (2022), Gao et al. (2022)	dNTP hydrolysis
NucC	NucC	Type III CBASS Type III CRISPR‐Cas	Lau et al. (2020)	Chromosomal DNA degradation
DUF1524	Endonuclease/Nicking endonuclease	GmrSD BrxU SspABCD‐SspE	Machnicka et al. (2015), Picton et al. (2021), Gao et al. (2022)	Endonuclease in BrxU and GmrSD Nickase in SspE
PF20469	Topoisomerase‐primase (TOPRIM) domain of OLD family nucleases	Gabija Wadjet PARIS1 PARIS2 Retron+TOPRIM	Gao et al. (2020), Cheng et al. (2021), Cheng et al. (2022), Rousset et al. (2022), Deep et al. (2022)	Nuclease
PF02646	RmuC domain	DISARM‐accessory component Shield	Macdonald et al. (2022)	Promiscuous nuclease activity
PF05099	Tellurite resistance protein TerB	Shango Bunzi	Millman et al. (2022), Johnson et al. (2022)	N/A
PF08378	Nuclease‐related domain (NERD)	Mokosh type I Nhi‐like	Millman et al. (2022)	N/A
PF13918	PLD‐like domain	Mokosh type I Mokosh type II Azaca	Millman et al. (2022)	N/A
PF06508	Queuosine biosynthesis protein QueC	qatABCD CBASS type IV	Gao et al. (2020), Millman et al. (2020)	Conversion of 7‐carboxy‐7‐deazaguanine (CDG) to 7‐cyano‐7‐deazaguanine (preQ_0_)
PF14455	Predicted metal binding domain assocated with E1, E2, and JAB domains	ISG15‐like CBASS type II	Ledvina et al. (2023), Millman et al. (2022)	Ubiquitin‐like system
PF14464	Prokaryotic homologues of the JAB domain	ISG15‐like CBASS type II	Ledvina et al. (2023), Millman et al. (2022)	Ubiquitin‐like system
PF14459	Prokaryotic E2 family C	ISG15‐like CBASS type II	Ledvina et al. (2023), Millman et al. (2022)	Ubiquitin‐like system
PF08937	Toll/interleukin‐1 (IL‐1) receptor (TIR)	Thoeris pycSAR CBASS type III Retron + TIR TIR‐NLR SPARTA(TIR‐APAZ and pAgo)	Gao et al. (2020), Tal et al. (2021), Ofir et al. (2021), Koopal et al. (2022), Rousset et al. (2022), Hogrel et al. (2022), Kibby et al. (2022)	Depletion of NAD+
PF02146	Silent information regulator 2 (Sir2)	Thoeris SPARSA(Sir2‐APAZ and pAgo) DSR1 DSR2 Sir2‐HerA Avast type V PD‐T7‐2	Gao et al. (2020), Ofir et al. (2021), Koopal et al. (2022), Garb et al. (2022)	For ThsA‐NADase activity produces signalling molecule for ThsB For DSR1 and DSR2 NADase activity leads to phage inhibition or cell death, respectively.
PF12770	CHAT Protease	Borvo CRISPR‐Cas Type III‐E	Liu et al. (2022), Millman et al. (2022)	In CRISPR‐Cas Type III‐E‐ Caspase‐like protease that cleaves Csx30, to induces an abortive infection
PF18735	HEPN	PD‐T4‐2 PD‐T4‐5 PD‐T7‐3 ApeA	Gao et al. (2020), Vassallo et al. (2022)	N/A
PF11645	PD(D/E)XK nuclease	PD‐T4‐1 PD‐T4‐4 PD‐T4‐8 PD‐T7‐1 PD‐T7‐5 PD‐λ‐3	Vassallo et al. (2022)	N/A
PF13555	P‐Loop NTPAse	PD‐T7‐2 PD‐T4‐4 PD‐λ‐4	Vassallo et al. (2022)	N/A
PF01541	GIY‐YIG nuclease	PD‐T4‐3 PD‐λ‐1	Vassallo et al. (2022)	N/A
PF18178	TIR‐ and PNP‐associating SLOG family	Cap17(CBASS type I and type III) Cap17(Detocs) Cap17(pAgo) Cap17(DRT8) Cap17(NLR‐like systems)	Rousset et al. (2023)	N/A
PF01048	Purine nucleoside phosphorylase (PNP)	Cap17(CBASS type I and type III) Cap17(Detocs) Cap17(pAgo) Cap17(DRT8) Cap17(NLR‐like systems)	Rousset et al. (2023)	Degradation of (d)ATP/cell death

*Note*: Where determined, the in vitro activity of each domain is indicated. Only domains that were predicted with a probability score higher than 50% are reported.

Finally, another example of a nuclease domain shared by distinct defence systems is the nuclease NucC, an effector protein first associated with several subtypes of the CBASS systems and is also found as accessory proteins in type III CRISPR‐Cas systems. In the CBASS system, upon activation by a cyclic second messenger, NucC was shown to assemble into homohexamers to elicit cleavage of double‐stranded DNA, leading to the depletion of bacterial chromosomal DNA and cell death (Lau et al., 2020). NucC homologues associated with type III CRISPR‐Cas systems can also induce cell death in response to jumbo phage infection (Mayo‐Muñoz et al., 2022).

Whilst predicted domains for antiphage genes frequently exhibit different PFAM identifiers, their predicted activity (e.g. AAA+ ATPase, nuclease and helicase) often remains similar (Table 1). These examples may therefore represent instances where an enzymatic activity such as DNA or RNA degradation was acquired independently, given the evolutionary advantage it may confer. Indeed, nucleic acid degradation can both provide the first line of defence by swiftly arresting phage infection but can also be used for simultaneous chromosome degradation, leading to the death of infected cells (Doron et al., 2018; Gao et al., 2020; Millman et al., 2022; Rousset et al., 2022; Vassallo et al., 2022). It must be also noted that in some cases, further phylogenetic and evolutionary analysis of frequently associated domains in defence proteins (i.e. DUF262‐carrying proteins with either a nuclease, TOPRIM or ATPase) could reveal distant but nevertheless phylogenetically‐linked families of defence proteins (Rousset et al., 2022). As some of these defence proteins may belong to the same family upon closer inspection, it is possible that they have diverged early in response to the evolutionary pressure posed by phage predation and counter‐defences.

Several studies suggest that AAA+ ATPase and helicase domains are involved in regulation and phage sensing (Cheng et al., 2021; Gao et al., 2020, 2022; Millman et al., 2022). Like nuclease domains, many predicted AAA+ ATPase and helicase domains are found in disparate antiphage system components, albeit only sometimes exhibiting the same PFAM identifier (Doron et al., 2018; Gao et al., 2020; Millman et al., 2022; Rousset et al., 2022; Vassallo et al., 2022) (Table 1). Similar to GajA, BrxU, SspE and GmrSD, other types of NTPase (AAA+ ATPase) domains may provide a sensing mechanism that regulates the downstream nuclease activity. Helicase domains of different families were also suggested to represent sensing modules in several newly‐discovered antiphage systems (Table 1) (Cheng et al., 2021; Gao et al., 2020, 2022; Millman et al., 2022). A complete mechanistic characterisation of many of these antiphage systems has yet to be performed, and therefore, the exact role these domains play, perhaps either as sensors or effectors, remains to be discovered.

#### 1.1.3 TIR and Sir2 domains

Toll/interleukin‐1 (IL‐1) receptor (TIR) domains and Silent information regulator 2 (Sir2) proteins, or sirtuins, are found in all domains of life (Wang et al., 2022). The recent spike in interest in bacterial immunity strategies led to the striking discovery that TIR and Sir2 domains can be involved in phage defence, and have been co‐opted by several different antiphage systems, leading, in all cases, to the programmed death of infected cells through the depletion of NAD+ (Wang et al., 2022).

TIR and Sir2 domains were first discovered in the antiphage system Thoeris, composed of ThsA (Sir2 domain) and ThsB (TIR domain). In this case, the Sir2 domain‐mediated NADase activity produces a signalling molecule that activates ThsB, which is responsible for NAD+ depletion through its TIR domain (Ofir et al., 2021).

In other instances, TIR and Sir2 domains are found separately as effector modules associated with other genes. This is the case for CBASS and pycSAR systems, wherein TIR domains are associated with nucleotide cyclases (Govande et al., 2021;Morehouse et al., 2020; Tal et al., 2021) and for the Retron+TIR system found by Gao et al., where the TIR‐harbouring component is associated with a reverse transcriptase (Gao et al., 2020). Upon production of a cyclic nucleotide that functions as a signal, cyclic‐di‐GMP for CBASS and cyclic‐UMP for pycSAR, TIR domains are activated and lead to cell death through NAD+ degradation (Morehouse et al., 2020; Tal et al., 2021). TIR domains are also associated with NACHT module‐containing proteins in bacteria. NACHT‐containing proteins are also part of the bacterial innate immunity arsenal against phages and display a tri‐modular structure with a central NACHT domain, a C‐terminal sensor and an N‐terminal effector region. In many cases, the effector region harbours a TIR or Sir2 domain, likely mediating abortive infection (Kibby et al., 2022).

Sir2 and TIR domain‐containing proteins are sometimes encoded next to prokaryotic argonautes (pAgos). These represent two antiphage systems: SPARTA (two‐partner system with TIR‐APAZ and pAgo) and SPARSA (two‐partner system with Sir2‐APAZ and pAgo). In SPARTA, invading nucleic acids are recognised by pAgos using guide RNA or DNA, causing the formation of SPARTA heterodimers and triggering the TIR‐APAZ NADase activity (Koopal et al., 2022).

Finally, Sir2 domains are found in Defence‐associated sirtuin systems DSR1 and DSR2, the Sir2‐HerA systems, PD‐T7‐2 and Avast type V (Gao et al., 2020; Garb et al., 2022; Vassallo et al., 2022). DSR2 unleashes NADase activity of the Sir2 domain upon recognition of a tail tube protein, leading to proposed abortive infection. Curiously, unlike other Sir2 and TIR‐containing antiphage systems, DSR1 inhibits phage replication without leading to cell death (Garb et al., 2022).

### 1.2 FINAL REMARKS

The studies discussed above highlight how different antiphage systems can sometimes co‐opt the same domains, resulting in a variety of different combinations. The frequent acquisition of certain domains by several distinct antiphage systems could denote an immediate fitness advantage conferred by that domain, presumably in response to phage assault.

TIR and Sir2 domains are found together, singularly or in combination with other domains in several defence systems. The examples discussed above suggest that several antiphage systems have independently acquired TIR and Sir2 domains, and thus their NADase activity likely represents an efficient strategy to combat phage infections. This is not surprising given the widespread distribution of Sir2 and TIR domains in all domains of life and the central roles that NAD+ or NADH play in maintaining cellular homeostasis (Wang et al., 2022). The recent reports that TIR domains of plant immune receptors can also catalyse NAD+ depletion further suggest that NAD+ depletion represents a widespread and efficient antiviral strategy that can quickly lead to death of the infected cells (Yu et al., 2022).

Aside from TIR and Sir2 domains, one of the most interesting but perhaps not surprising observations is the high number of antiphage systems that harbour at least one component with a predicted nuclease or nicking activity, many of which share the same PFAM identifier (Doron et al., 2018; Rousset et al., 2022; Vassallo et al., 2022) (Table 1). From an evolutionary point of view, acquisition of the same domain by antiphage systems could represent a disadvantage, facilitating the evolution of phage counter‐measures. However, it is also easy to envision how an effector protein that alters or degrades DNA and RNA represents one of the most efficient means to wipe out a phage infection, whether this is provided through degradation of phage nucleic acids or simultaneous cleavage of phage and bacterial nucleic acids, to cause the death of infected cells. As reported above, BrxU, GmrSD and SspE all contain similar components but exhibit different specificities either for NTPs or in their nuclease activities. This variation ultimately leads to broader protection against a more diverse range of phages (He et al., 2015; Picton et al., 2021; Xiong et al., 2020).

Indeed, we can now readily observe how multiple factors combine to diversify the spectrum of targeted phages and potentially reduce the development of phage counter‐defences. This now includes the frequent recurrence of some nuclease‐like domains in many different antiphage systems, the variation in the combination of sensing and effector modules, the different specificity of the sensing modules for cyclic nucleotide signals, NTPs or phage factors and differences in the fold and activity of the effector modules. No doubt as efforts continue towards expanding the discovery and characterisation of bacterial immunity systems we will increasingly note functional overlaps, which may in turn help to broaden our understanding of immunity in higher organisms.

## AUTHOR CONTRIBUTIONS

Giuseppina Mariano: Writing – original draft; Writing – review & editing; Conceptualization. Tim R. Blower: Writing – original draft; Writing – review & editing.

## FUNDING INFORMATION

This work was supported by a Wellcome Trust Sir Henry Wellcome Fellowship (218622/Z/19/Z) to G.M. and by a Lister Institute Prize Fellowship to T.R.B.

## ETHICS STATEMENT

No human or animal subjects were used in this study.

## CONFLICT OF INTEREST STATEMENT

The authors declare that there are no conflicts of interest.

## Data Availability

Data sharing not applicable to this article as no datasets were generated or analysed during the current study.
